# Structural Expansion of Catalytic RNA Nanostructures through Oligomerization of a Cyclic Trimer of Engineered Ribozymes

**DOI:** 10.3390/molecules28186465

**Published:** 2023-09-06

**Authors:** Mst. Ayesha Siddika, Hiroki Oi, Kumi Hidaka, Hiroshi Sugiyama, Masayuki Endo, Shigeyoshi Matsumura, Yoshiya Ikawa

**Affiliations:** 1 Graduate School of Innovative Life Science, University of Toyama, Toyama 930-8555, Toyama, Japansmatsumu@sci.u-toyama.ac.jp (S.M.); 2 Department of Chemistry, Graduate School of Science and Engineering, University of Toyama, Toyama 930-8555, Toyama, Japan; 3 Department of Chemistry, Graduate School of Science, Kyoto University, Kyoto 606-8501, Kyoto, Japan; 4 Institute for Integrated Cell-Material Sciences, Kyoto University, Kyoto 606-8501, Kyoto, Japan; sugiyama.hiroshi.3s@kyoto-u.ac.jp (H.S.); endo@kansai-u.ac.jp (M.E.); 5 Organization for Research and Development of Innovative Science and Technology, Kansai University, Suita 564-8680, Osaka, Japan

**Keywords:** group I, ribozyme, RNA motif, RNA nanostructure, *Tetrahymena*

## Abstract

The multimolecular assembly of three-dimensionally structured proteins forms their quaternary structures, some of which have high geometric symmetry. The size and complexity of protein quaternary structures often increase in a hierarchical manner, with simpler, smaller structures serving as units for larger quaternary structures. In this study, we exploited oligomerization of a ribozyme cyclic trimer to achieve larger ribozyme-based RNA assembly. By installing kissing loop (KL) interacting units to one-, two-, or three-unit RNA molecules in the ribozyme trimer, we constructed dimers, open-chain oligomers, and branched oligomers of ribozyme trimer units. One type of open-chain oligomer preferentially formed a closed tetramer containing 12 component RNAs to provide 12 ribozyme units. We also observed large assembly of ribozyme trimers, which reached 1000 nm in size.

## 1. Introduction

Hierarchical folding and assembly are characteristic features of biomacromolecules required to exhibit their functions in living systems. The defined tertiary structures of single polypeptide proteins are usually composed of smaller and simpler structural elements, such as α-helices and β-sheets. Secondary structural elements are then assembled into globular tertiary structures, which are required for biological functions. Some structured proteins further assemble to yield their quaternary structures, which enable sophisticated and complex functions impossible for their monomer subunits without assembly.

Hemoglobin is an extensively studied example of protein assembly and its accompanied functional development [1,2]. The subunit of hemoglobin (globin subunit), which is highly similar to myoglobin, forms a tetramer in vertebrates. Assembly of globin subunits is much more diverse in invertebrates, with the number of subunits assembled ranging from 2 to 144 [1,2,3,4,5]. The hemoglobin of the deep-sea tubeworm *Rifitia pachyptila* is assembled from 24 globin subunits, which can be dissected into a pair of globin dodecamers [4,5,6]. A similar structure of a globin dodecamer is also found in giant hemoglobin (erytrocruorin), which contains 144 globin subunits forming 12 globin dodecamer units [4,6]. These globin dodecamers can be further dissected into three globin tetramers [4,5,6]. The assembly mode of this tetramer unit is similar to a tetrameric hemoglobin from mollusk, which has a distinct assembly mode from vertebrate tetrameric hemoglobin.

Hierarchical folding of open-chain biopolymers to form defined tertiary structures is also observed in RNA molecules [7,8]. In the hierarchical formation of RNA tertiary structures, open-chain RNAs first form secondary structures composed of Watson–Crick duplexes (stem elements) [7,8]. RNA secondary structures then fold into RNA tertiary structures supported by multiple long-range tertiary interactions [9,10,11]. These RNA tertiary structures exhibiting biological functions have evolved in nature or can be produced in vitro. In contrast to protein quaternary structures, which often show high structural symmetry, natural evolution of structured RNAs has poorly exploited the corresponding quaternary structures with structural symmetry.

**Figure 1:**
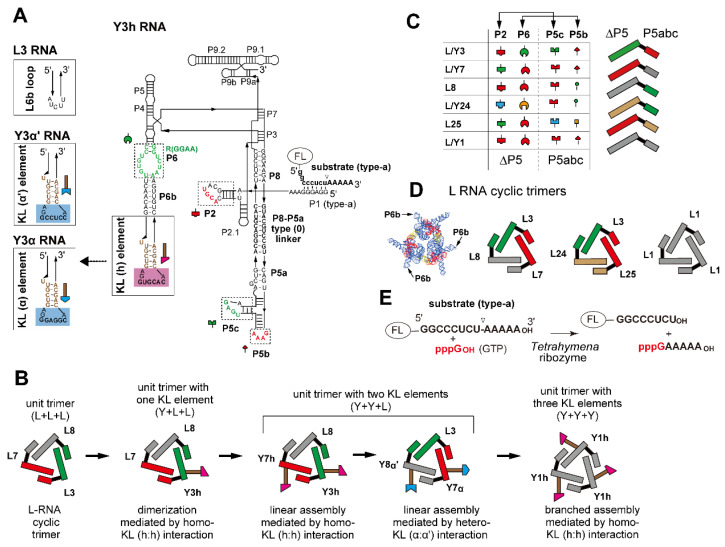
Design of ribozyme cyclic trimers as unit modules with oligomerization ability. (**A**) Secondary structure of engineered *Tetrahymena* ribozyme (Y3h RNA). Boxes with solid lines indicate the terminal region of the P6b element to which the kissing loop (KL) unit was introduced. L3 RNA had the original P6b element. Boxes with broken lines indicate modularly replaceable RNA elements to derive variants from the parent L3 RNA. Broken lines with arrowheads indicate RNA motifs used to design variants of Y3α and Y3α′ RNAs. Nucleotides shown in red constitute RNA motifs responsible for the intermodule tertiary interactions and present in the *Tetrahymena* ribozyme, L1 RNA, and Y1h RNAs. (**B**) Scheme of stepwise modular design of ribozyme cyclic trimers with homo- or hetero-oligomerization ability. (**C**) Tertiary interactions constituting interfaces between the ∆P5 ribozyme module and the P5abc module (left). L RNAs possessing distinct sets of ∆P5 and P5abc modules (right). Cognate pairs of ∆P5 and P5abc modules to form matched ∆P5/P5abc interfaces are shown in the same colors. (**D**) Three L RNA cyclic trimers used in this study. The L3, L7, and L8 trio and the L3, L24, and L25 trio formed heterotrimers, while L1 RNA formed a homotrimer. A model three-dimensional (3D) structure of L RNA trimer is also shown on the left. (**E**) The substrate cleavage reaction promoted by the *Tetrahymena* ribozyme and its engineered variants. The fluorophore FAM or TAMRA was attached to the 5′-end of the substrate RNA to visualize its cleavage.

To complement the natural evolution of structured RNAs, RNA quaternary structures with structural symmetry have been rationally designed [12,13,14,15]. We also rationally engineered two classes of large unimolecular ribozymes (*Tetrahymena* group I intron ribozyme and bacterial ribonuclease P ribozyme) to afford oligomerization ability to these ribozymes without sacrificing their catalytic ability [16,17,18]. Rational engineering of the *Tetrahymena* ribozyme tertiary structure was performed in a two-step manner [16]. In the first step, a large structural module (P5abc), which extends from the P5 element and binds to the core ribozyme module (∆P5 RNA), was physically separated to yield a bimolecular form of the ribozyme (Figure S1), in which the two RNAs folded independently and formed a highly stable and catalytically active complex (P5abc/∆P5 bimolecular ribozyme) (Figure S1) [19,20,21]. In the second step, the physically separated P5abc module was joined to the P8 element of the core ∆P5 ribozyme via a rigid duplex linker (P8-P5a linker in Figure 1A) to yield a class of derivatives of the *Tetrahymena* ribozyme (L1 RNA and a series of variants termed L RNAs) (Figure S1) [16]. The P5abc module in L1 RNA interacted exclusively with the ∆P5 module in an intermolecular manner and induced homo-oligomerization of L1 RNA (L/L/L cyclic trimer) (Figure S1). Through optimization of the P8-P5a linker element, L1 RNA preferentially formed a closed form (cyclic) homotrimer and small amounts of cyclic homotetramer, the structures of which were confirmed by atomic force microscopy (AFM) [16]. Engineering of the RNA–RNA recognition interface between the P5abc module and ∆P5 module yielded a trio of L1 RNA variants (L3, L7, and L8) (Figure 1) and also a pair of other variants (L2 and L3). These variants were designed for selective formation of a cyclic heterotrimer (L3/L7/L8) and a cyclic heterotetramer (L2/L3/L2/L3), respectively [16]. These cyclic oligomers may be regarded as unit oligomers like hemoglobin tetramers that allow the formation of higher oligomeric states (such as hemoglobin dodecamers and giant hemoglobin) in a modular manner.

This study was performed to develop ribozyme assemblies larger than the above trimer and tetramer in a stepwise manner. By mimicking the development of giant hemoglobin, we regarded the ribozyme trimers (L3/L7/L8 and L1/L1/L1) as unit oligomers corresponding to the globin tetramer unit in giant hemoglobin. To oligomerize the unit trimers, we introduced kissing loop (KL) interaction motifs [22] as oligomerization units (Figure 1A,B and Figure S2). The resulting variants (termed Y RNA in this study) were examined as components of the unit trimers. We found that the unit trimer consisting of two Y RNAs and one L RNA (L/Y/Y-type trimers) preferentially formed cyclic tetramers containing 12 L/Y RNAs. The resulting ribozyme dodecamer may be compared with the globin dodecamer in giant hemoglobin.

## 2. Results

### 2.1. Introduction of One KL Motif in a Ribozyme Trimer for Homodimerization

**Figure 2:**
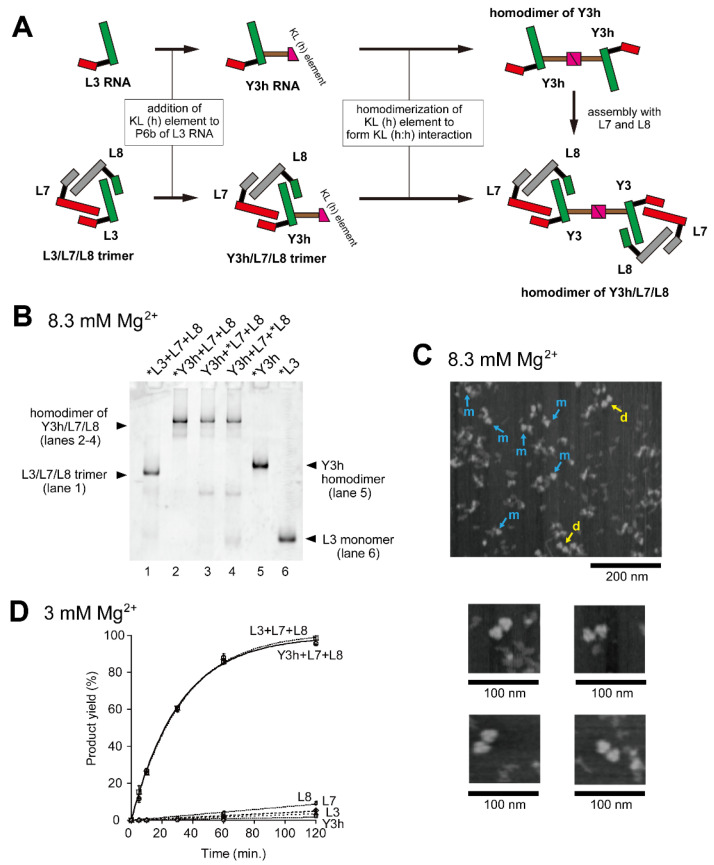
Homodimerization of the Y3h/L7/L8 trimer. (**A**) Modular engineering of the L3/L7/L8 trimer to construct its homodimer through introduction of the homo-KL (h:h) interaction. (**B**) EMSA of the L3/L7/L8 trimer and the Y3h/L7/L8 trimer in Tris-borate buffer (pH 8.3) containing 8.3 mM Mg^2+^. In lanes 1 and 2–4, the concentration of each RNA component was 0.40 μM. In lanes 5 and 6, the concentration of the RNA component was 0.60 μM. Asterisks indicate monomer RNAs labeled with BODIPY fluorophore. (**C**) AFM imaging of the Y3h/L7/L8 trimer and its homodimer. In the top image, objects corresponding to the Y3h/L7/L8 trimer and its homodimer are indicated by the blue arrows marked with m and yellow arrows marked with d, respectively. Each of four smaller images shows the homodimer of the Y3h/L7/L8 trimer. AFM was performed in Tris-borate buffer (pH 8.3) containing 8.3 mM Mg^2+^. (**D**) Substrate cleavage reactions by the L3/L7/L8 trimer, Y3h/L7/L8 trimer, and their component RNAs. The six reactions commonly contained 0.90 μM substrate-a. In the reaction with L3, L7, L8, or Y3h, the concentration of ribozyme RNA was 0.90 μM. In the reaction with L3 + L7 + L8 or Y3h + L7 + L8 RNA, the concentration of each ribozyme component was 0.30 μM. Reactions were carried out in the presence of 3 mM Mg^2+^.

We first designed a dimeric form of a closed L RNA trimer by introducing a KL interaction to one of three L RNA units in each trimer (Figure 1 and Figure S1). The stem-loop element for the KL interaction was introduced at the P6b element in L RNA (Figure 1 and Figure 2A). The resulting hexamer (dimer of Y/L/L trimers) formed a dumbbell shape (Figure S1). We employed a heterotrimer consisting of L3, L7, and L8 RNAs as a parent L RNA closed trimer (Figure 1C,D) because it formed a closed heterotrimer both efficiently and selectively [16]. We introduced a homodimeric KL element that was identified as the dimerization initiation site (DIS) of HIV-1 genomic RNA [23]. This motif self-dimerizes to form a homo-KL interaction, in which two hairpin loops are assembled nearly coaxially (Figure S3) [22,23]. We prepared a variant of L3 RNA possessing the HIV-DIS motif to the P6b element (Figure 1A). A series of variant L RNAs with KL elements in P6b was designated as Y RNA, and the parent HIV-DIS homo-KL motif (subtype A) (Figure S3) and their dimeric interaction are referred to as “h” and “h:h”, respectively, in this study. A variant L3 RNA was thus designated as Y3h RNA. Y3h RNA was predicted to form a homodimer with the KL (h:h) interaction (Figure 1A and Figure 2A).

In the presence of 8.3 mM Mg^2+^, homodimerization of Y3h RNA was experimentally confirmed by an electrophoretic mobility shift assay (EMSA) (Figure 2B, lane 5), in which the mobility of Y3h RNA was distinctly slower than that of the parent L3 RNA. L3 RNA formed a monomeric state in the absence of its partner RNAs (Figure 2B, lane 6). No homodimerization of Y3h RNA was seen without Mg^2+^, under which conditions the mobility of Y3h RNA is close to that of L3 RNA (Figure S4, lanes 1 and 2). This observation was consistent with a previous report that the formation of the KL (h:h) interaction requires Mg^2+^ [24,25,26] and suggested that Y3h existed in a monomeric state. Stable formation of the KL (h:h) interaction was also observed in the Y3h/L7/L8 ribozyme trimer because it showed slower mobility than the L3/L7/L8 trimer (Figure 2B, lanes 1–4). L3/L7/L8 trimer formation, Y3h/L7/L8 trimer formation, and dimerization of Y3h/L7/L8 trimer were not observed in the absence of Mg^2+^ (Figure S4, lanes 5 and 6). These observations were also consistent with the requirement for Mg^2+^ for assembly of the P5abc and ∆P5 modules [16,27].

To obtain direct evidence of dimerization of the Y3/L7/L8 trimer through the KL (h:h) interaction, we performed AFM measurement of samples containing Y3h, L7, and L8 RNAs (Figure 2C). In the presence of 8.3 mM Mg^2+^, triangular objects corresponding to Y3h/L7/L8 trimers were observed (Figure 2C, blue arrows marked with m and yellow arrows marked with d). We also obtained images of two trimers located in close contact (Figure 2C, yellow arrows marked with d). No such images were observed on AFM analysis of L3/L7/L8 trimers reported previously [16]. These observations strongly suggested that two trimers located in close contact represented homodimers of Y3h/L7/L8 trimers supported by the KL (h:h) interaction.

To determine the effect of homodimerization of the ribozyme trimers, we also analyzed their substrate cleavage activity (Figure 1E). In the presence of 3 mM Mg^2+^, each RNA component (L3, L7, L8, or Y3h RNA) was nearly inactive (Figure 2D). These results were consistent with the previous observation that the core catalytic module (∆P5 ribozyme) was nearly inactive in the presence of 3 mM Mg^2+^. In the presence of a trio of L RNAs (L3, L7, and L8), distinct catalytic activity was observed with a reaction rate of 0.030 min^−1^ (Figure 2D). This suggested L3/L7/L8 heterotrimer formation, in which the core P5 modules in L RNAs were activated by the association with the P5abc module provided by the partner RNA. In the presence of Y3h, L7, and L8, the efficiency of the substrate cleavage reaction (reaction rate: 0.032 min^−1^) was similar to that with L3/L7/L8 (0.030 min^−1^) (Figure 2D), suggesting that dimerization of the ribozyme-trimer has no effect on its catalytic function.

### 2.2. Introduction of Two KL Motifs in a Ribozyme Trimer for Oligomerization

**Figure 3:**
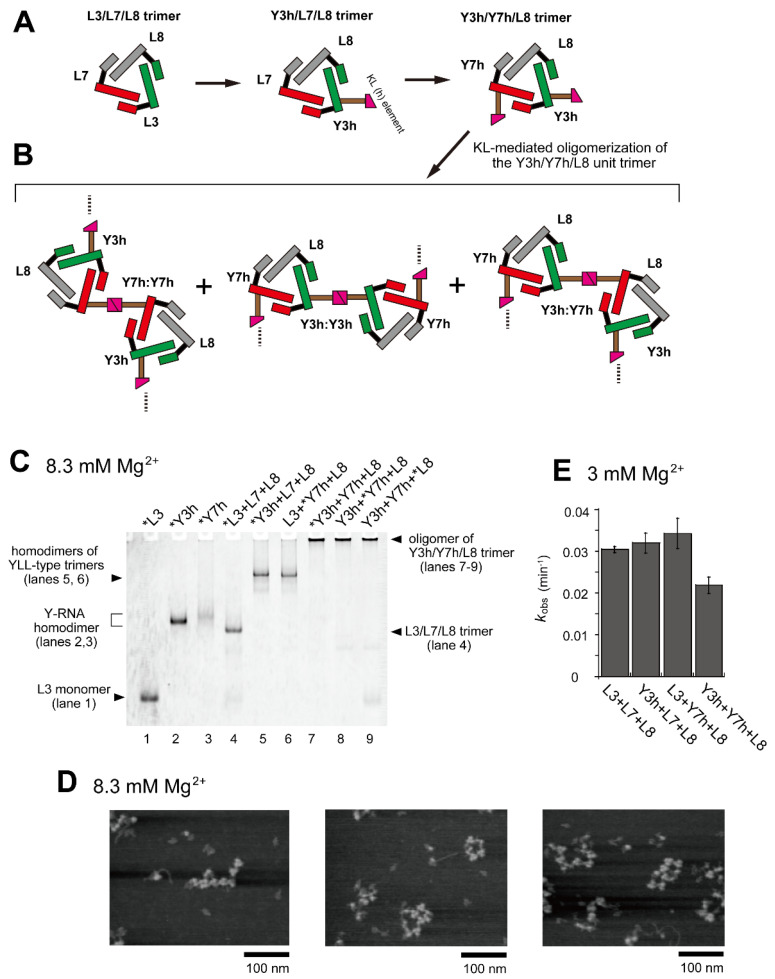
Oligomerization of the Y3h/Y7h/L8 trimer. (**A**) Modular engineering of the L3/L7/L8 trimer to construct its oligomer through introduction of the homo-KL (h:h) interaction. (**B**) Three distinct modes of dimerization of the unit trimers (Y3h/Y7h/L8) by KL (h:h) interaction. (**C**) EMSA of the L3/L7/L8 trimer and Y3h/Y7h/L8 trimer in Tris-borate buffer (pH 8.3) containing 8.3 mM Mg^2+^. In lanes 1–3, the concentration of the RNA component was 0.60 μM. In lanes 4–9, the concentration of each RNA component was 0.40 μM. Asterisks indicate monomer RNAs labeled with BODIPY fluorophore. (**D**) AFM imaging of homo-oligomers of the Y3h/Y7h/L8 unit trimer. AFM was performed in Tris-borate buffer (pH 8.3) containing 8.3 mM Mg^2+^. (**E**) Substrate cleavage reactions by the parent ribozyme trimer, its dimers, and its oligomer. The four reactions contained 0.90 μM substrate-a. In each reaction, the concentration of each ribozyme component was 0.30 μM. Reactions were carried out in the presence of 3 mM Mg^2+^.

Based on the observation that the introduction of a single homo-KL motif element for homo-KL (h:h) interaction to the L3/L7/L8 ribozyme trimer (Y3h/L7/L8) resulted in its homodimerization, we then introduced the homo-KL (h) motif element into two of three component L RNAs. We prepared Y7h RNA to form a unit trimer (Y3h/Y7h/L8) bearing two homo-KL elements in the unit (Figure 1B and Figure 3A). The Y3h/Y7h/L8 trimer was predicted to self-oligomerize although its oligomerization would be poorly ordered (Figure 3B). We first confirmed that homodimerization of L3/L7/L8 trimer was also induced by the introduction of the KL (h) element to L7 RNA (Figure 3C, lane 6).

In EMSA, RNA samples containing a set of RNAs (Y3h, Y7h, and L8) were stuck in the sample wells (Figure 3C, lanes 7–9), suggesting that the trio of RNAs formed oligomeric states containing more than eight L and/or Y RNA units because previous EMSA experiments showed that gel mobility of two types of L RNA octamers were low but detectable [28,29]. The extent of Y3h/Y7h/L8 unit trimer oligomerization was also investigated by AFM (Figure 3D). Various forms of oligomers of the Y3h/Y7h/L8 unit trimer or its components were observed. Most appeared to form open-chain oligomers of the unit trimer. We also investigated the catalytic activity of the set of RNAs (Y3h/Y7h/L8) (Figure 3E). The activities of two KL-mediated homodimers of cyclic trimers (homodimer of Y3h/L7/L8 and homodimer of L3/Y7h/L8) exhibited no negative effect on ribozyme activity. The activity of the Y3h/Y7h/L8 trimer (0.022 min^−1^), however, was 1.36-fold lower than that of the parent L3/L7/L8 trimer (0.030 min^−1^).

### 2.3. Directional Assembly of Ribozyme Trimers by Hetero-KL Interactions

Introduction of two KL (h) motif elements to the L3/L7/L8 trimer produced oligomers, and the oligomerization of the resulting trimer (Y3h/Y7h/L8) was supported by the homo-KL (h:h) interaction (Figure 3C,D). While the homo-KL interaction efficiently induced assembly of the unit trimers, the directionality of the assembly was poorly controlled (Figure 3B,D). To improve the directionality of KL-mediated assembly, we substituted the KL (h:h) interaction with variants that formed hetero-KL interactions (Figure S2F). We designed and prepared two pairs of hetero-KL interactions designated as α:α′ pair and β:β′ pair (Figure S2F), which were derived from subtype A and subtype B DIS (Figure S3), respectively [23]. We first substituted the KL (h) motif element in Y3h with the hetero-KL motif elements α and α′ to yield a pair of Y3 RNAs (Y3α RNA and Y3α′ RNA) (Figure 1A).

**Figure 4:**
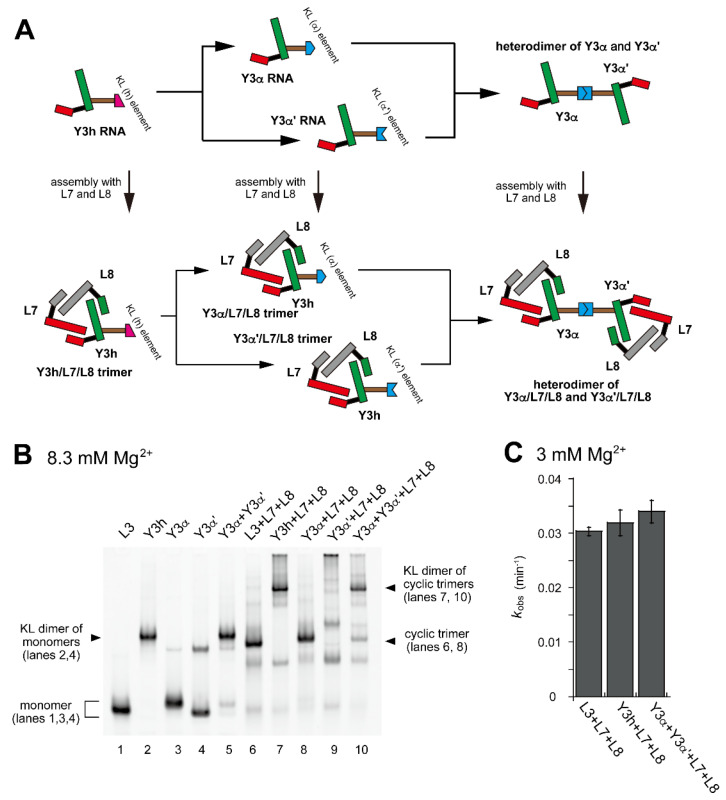
Dimerization of a pair of ribozyme cyclic trimers through hetero-KL interaction. (**A**) Modular engineering to dimerize the L3/L7/L8 trimer to a pair of ribozyme cyclic trimers that formed a dimer through a hetero-KL (α:α′) interaction. (**B**) EMSA of the L3/L7/L8 trimer and its derivatives to form a heterodimer of the cyclic trimer in Tris-borate buffer (pH 8.3) containing 8.3 mM Mg^2+^. In lanes 1–4, the concentration of the RNA component was 0.60 μM. In lane 5, the concentration of each RNA component was 0.30 μM. In lanes 6–9, the concentration of each RNA component was 0.40 μM. In lane 10, the concentration of Y3α and Y3α′ RNA was 0.20 μM and the concentration of L7 and L8 RNA was 0.40 μM. (**C**) Substrate cleavage reactions by the parent ribozyme trimer, its dimers, and its oligomer. The four reactions contained 0.90 μM substrate-a. In the reactions with three RNA components, the concentration of each ribozyme component was 0.30 μM. In the reaction with four RNA components, the concentration of Y3α and Y3α′ RNA was 0.15 μM and the concentration of L7 and L8 RNA was 0.30 μM. Reactions were carried out in the presence of 3 mM Mg^2+^.

In EMSA with 8.3 mM Mg^2+^, electrophoretic mobilities of Y3α RNA and Y3α′ RNA were similar to that of L3 RNA (Figure 4B, lanes 1, 3 and 4), indicating that Y3α and Y3α′ predominantly migrated as a monomer. Under the same conditions, Y3α′ RNA exhibited the second band, the intensity and mobility of which were lower than those of the monomer band (Figure 4B, lane 4). A similar second band was also observed for Y3α RNA, although its signal intensity was very weak (Figure 4B, lane 3). The unpredicted second band of Y3α′ RNA was presumably caused by unexpected interactions inducing its self-dimerization. Possible self-dimers of Y3α′ RNA, however, were resolved in the presence of the cognate partner of KL interaction (Y3α RNA), with which Y3α′ RNA yielded a heterodimer mediated by the hetero-KL (α:α′) interaction (Y3α/Y3α′) (Figure 4B, lane 5). The mobility of the heterodimer was nearly the same as that of the homodimer of Y3h RNA (Figure 4B, lanes 2 and 5).

In the presence of L7 and L8 RNAs, Y3α and Y3α′ were expected to form ribozyme trimers (Figure 4A). Assembly of Y3α, L7, and L8 formed a dominant band with slightly lower mobility on EMSA than the L3/L7/L8 trimer (Figure 4B, lane 8). Assembly of Y3α′, L7, and L8 formed a major band that remained stuck in the sample well (Figure 4B, lane 9), suggesting that the Y3α′/L7/L8 trimer underwent further oligomerization due presumably to Y3α′ RNA. The KL (α:α′) interaction was expected to induce heterodimerization of two ribozyme trimers. Assembly of Y3α/L7/L8 and Y3α′/L7/L8 trimers formed a major band (Figure 4B, lane 10) with similar mobility to Y3h/L7/L8 homodimer (Figure 4B, lanes 4 and 7). This observation suggested that the KL (α:α′) interaction mediated the heterodimer formation of ribozyme trimers and also showed that unexpected oligomerization of Y3α′/L7/L8 trimer (Figure 4B, lane 9) was solved by the formation of the cognate α:α′ KL interaction between Y3α and Y3α′ in the two ribozyme trimers. Consistent with homodimerization of the L3/L7/L8 trimer unit by the homo-KL (h:h) interaction, dimerization of the L3/L7/L8 trimer unit by the hetero-KL (α:α′) interaction also did not significantly affect the ribozyme activity (Figure 4C).

**Figure 5:**
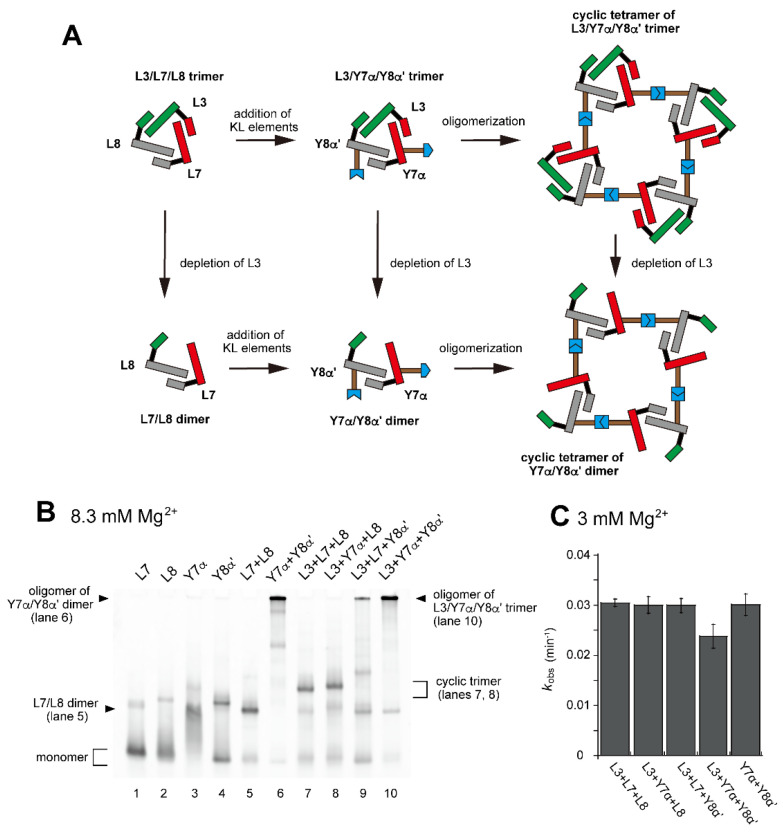
Preferable tetramerization of a ribozyme cyclic trimer through a hetero-KL (α:α′) interaction. (**A**) Modular engineering to oligomerize the L3/L7/L8 trimer through introduction of the hetero-KL (α:α′) interaction. The α and α′ elements were introduced into L7 RNA and L8 RNA to yield Y7α and Y8α′, respectively. (**B**) EMSA of the L3/L7/L8 trimer, its derivatives, and components to form oligomers of the cyclic trimer in Tris-borate buffer (pH 8.3) containing 8.3 mM Mg^2+^. In lanes 1–4, the concentration of the RNA component was 1.2 μM. In lanes 5 and 6, the concentration of each RNA component was 0.60 μM. In lanes 7–10, the concentration of each RNA component was 0.40 μM. (**C**) Substrate cleavage reactions by the parent ribozyme trimer, its derivatives, and their components. The five reactions contained 0.90 μM substrate-a. In the reactions with three RNA components, the concentration of each ribozyme component was 0.30 μM. In the reaction with two RNA components, the concentration of each ribozyme component was 0.45 μM. Reactions were carried out in the presence of 3 mM Mg^2+^.

Based on the introduction of a single α:α′ KL pair to the L3/L7/L8 trimer, resulting in its heterodimerization, we next introduced two hetero-KL pairs into one L3/L7/L8 trimer unit (Figure 5A). We introduced a KL (α) element into L7 RNA to yield Y7α RNA (Figure 5A and Figure S5). We also introduced the KL (α′) element as the partner of the KL (α) element into L8 RNA to yield Y8α′ RNA (Figure 5A and Figure S5). Assembly of L3, Y7α, and Y8α′ RNAs would yield a trimer L3/Y7α/Y8α′ (Figure 5A). The L3/Y7α/Y8α′ trimer served as a unit that would further oligomerize to yield oligomeric states of L3/L7/L8-type trimer units. The assembly state of L3/Y7α/Y8α′ would be similar to but more ordered than that of Y3h/Y7h/L8 trimer because of the distinct KL interactions (hetero-KL in α:α′ vs. homo-KL in h:h).

**Figure 6:**
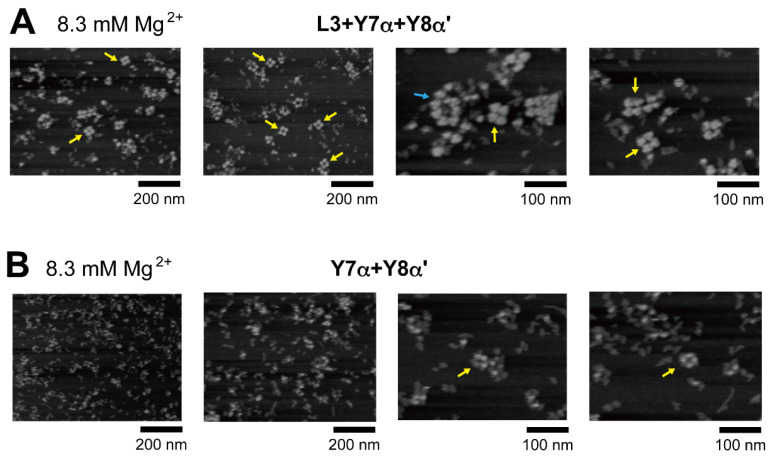
AFM imaging of the cyclic tetramer of the ribozyme cyclic trimer units. (**A**) AFM imaging of oligomeric states of the L3/Y7α/Y8α′ trimer. The cyclic tetramers of the L3/Y7α/Y8α′ trimer are indicated by the yellow arrows. An open-form octamer of the trimer is indicated by the blue arrow. AFM was performed in Tris-borate buffer (pH 8.3) containing 8.3 mM Mg^2+^. (**B**) AFM imaging of oligomeric states of the Y7α/Y8α′ dimer. Possible cyclic tetramers of the Y7α/Y8α′ dimer are indicated by the yellow arrows. AFM was performed in Tris-borate buffer (pH 8.3) containing 8.3 mM Mg^2+^.

In the presence of three RNA components (L3, Y7α, and Y8α′) and 8.3 mM Mg^2+^, oligomeric states of ribozyme trimers were observed in EMSA and AFM images (Figure 5 and Figure 6). While EMSA afforded no information regarding the number of component RNAs (Figure 5B, lane 10), AFM images indicated that the structural variety of oligomers was less diverse in the L3/Y7α/Y8α′ trio than in the Y3h/Y7h/L8 trio (Figure 6A). In AFM images of oligomers of the L3/Y7α/Y8α′ trimer, cyclic tetramers (consisting of 12 component RNAs) were frequently observed (Figure 6A, yellow arrows). Open-form oligomers of the L3/Y7α/Y8α′ trimer were also observed, some of which appeared to be octamers of the unit trimer, meaning that this oligomer contained 24 component RNAs (Figure 6A, blue arrow). EMSA indicated that the KL (α:α′) interaction-dependent oligomerization of Y7α and Y8α′ occurred even in the absence of L3 RNA (Figure 5B, lane 6). We directly observed AFM images of the sample solution containing Y7α and Y8α′ but not L3 to determine the contribution of Y3 RNA to the preferred formation of the closed tetramer of the L3/Y7α/Y8α′ unit trimer (Figure 6B). In the absence of L3 RNA, oligomeric forms of Y7α and Y8α′ were observed (Figure 6B). Oligomerization of Y7α and Y8α′ appeared less controlled than that of L3/Y7α/Y8α′. In AFM images, a possible tetramer of the Y7α/Y8α′ unit dimer was observed, but at much lower frequency than the tetramer of the L3/Y7α/Y8α′ unit trimer.

We also compared the activity of the oligomerization-competent set of RNAs (L3/Y7α/Y8α′) with the parent sets (L/L/L-type and Y/L/L-type) (Figure 5C). The activity of the L3/Y7α/Y8α′ set (0.024 min^−1^) was slightly lower than that of the parent sets (0.029–0.03 min^−1^). The activity of L3/Y7α/Y8α′ set (0.024 min^−1^) was similar to that of the Y3h/Y7h/L8 set (0.022 min^−1^) (Figure 3E), oligomerization of which was mediated by homo-KL interaction. The oligomerization-competent pair of Y RNAs (Y7α and Y8α′) showed nearly the same activity (0.029 min^−1^) as the parent trimer (L3/L7/L8, 0.030 min^−1^) even without L3 RNA (Figure 5C).

**Figure 7:**
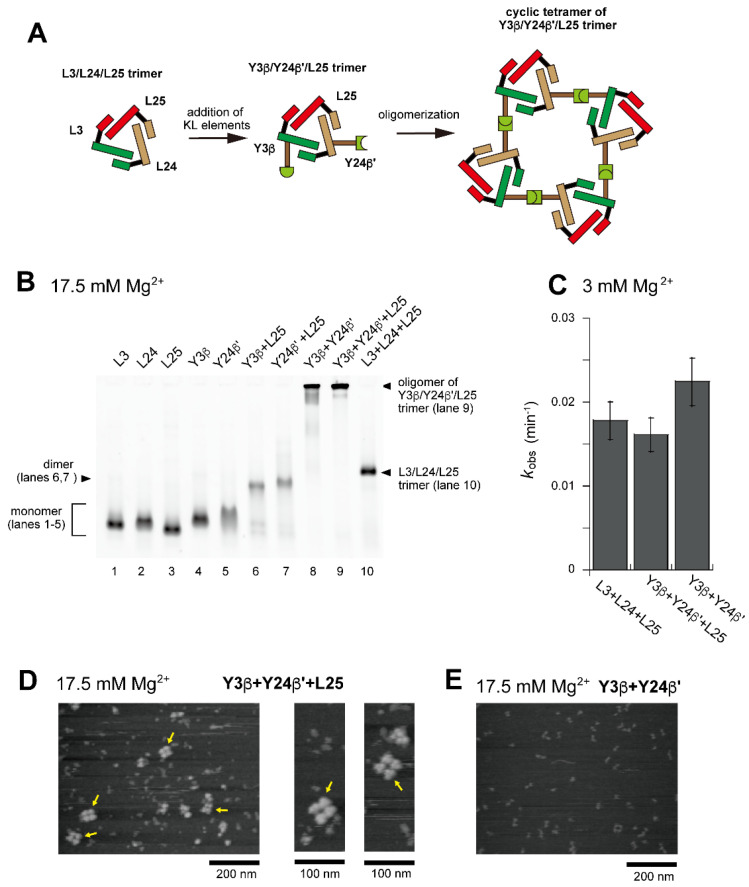
Preferential tetramerization of a ribozyme cyclic trimer through a hetero-KL (β:β′) interaction. (**A**) Modular engineering to oligomerize the L3/L24/L25 trimer through introduction of the hetero-KL (β:β′) interaction. The β and β′ element were introduced into L3 RNA and L24 RNA to yield Y3β and Y24β′, respectively. (**B**) EMSA of the L3/L24/L25 trimer, its derivatives, and components to form oligomers of the cyclic trimer in Tris-acetate buffer (pH 7.5) containing 17.5 mM Mg^2+^. In lanes 1–5, the concentration of the RNA component was 1.2 μM. In lanes 6–8, the concentration of each RNA component was 0.60 μM. In lanes 9 and 10, the concentration of each RNA component was 0.40 μM. (**C**) Substrate cleavage reactions by the parent ribozyme trimer, its derivatives, and their components. The five reactions contained 0.90 μM substrate-a. In the reactions with three RNA components, the concentration of each ribozyme component was 0.30 μM. In the reaction with two RNA components, the concentration of each ribozyme component was 0.45 μM. Reactions were carried out in the presence of 3 mM Mg^2+^. (**D**) AFM imaging of oligomeric states of the Y3β/Y24β′/L25 trimer. The cyclic tetramers of the Y3β/Y24β′/L25 trimer are indicated by the yellow arrows. AFM was performed in Tris-acetate buffer (pH 7.5) containing 17.5 mM Mg^2+^. (**E**) AFM imaging of oligomeric states of the Y3β/Y24β′ dimer. AFM was performed in Tris-acetate buffer (pH 7.5) containing 17.5 mM Mg^2+^.

We next investigated the assembly of a tetramer of the unit ribozyme trimer by changing both the ribozyme trimer and the hetero-KL interaction. As the second trio of L RNAs to form a unit ribozyme trimer, we used L3, L24, and L25 (Figure 1D, Figure 7A and Figure S5). They also assembled to form the L3/L24/L25 cyclic trimer (Figure 7B, lane 10). As an alternative hetero-KL interaction, we used the β:β′ KL pair (Figure S2F). EMSA was performed in the presence of 17.5 mM Mg^2+^ because the L3/L24/L25 trimer with three ∆P5/P5abc interfaces was less stable than the L3/L7/L8 trimer and the stability of ∆P5/P5abc interfaces was shown to be improved by increasing Mg^2+^ concentration [27,30]. Although EMSA provided insight into either detailed compositions or structures, the sample solution containing Y3β, Y24β′, and L25 afforded a dominant band that remained stuck in the sample well, indicating the formation of an oligomeric state (Figure 7B, lane 9). The substrate cleavage activity of the L3/L24/L25 trimer (0.018 min^−1^) (Figure 7C) was lower than that of the L3/L7/L8 trimer (0.030 min^−1^) (Figure 3E). Installation of the oligomerization ability by hetero-KL (β:β′) interaction appeared to cause a slight reduction of the activity (0.016 min^−1^) (Figure 7C). With the same sample solution, AFM yielded images corresponding to cyclic tetramers of the Y3β/Y24β′/L25 trimer, which were similar to the cyclic tetramers of the L3/Y7α/Y8α′ trimer (Figure 7D). We also examined oligomerization of Y3β and Y24β′ in the absence of L25. While the sample solution of Y3β and Y24β′ formed a band that remained stuck in the sample wells on EMSA (Figure 7B, lane 8), oligomers were hardly observed in AFM measurement (Figure 7E).

### 2.4. Large Assembly Structure Formed by Single RNA Component

**Figure 8:**
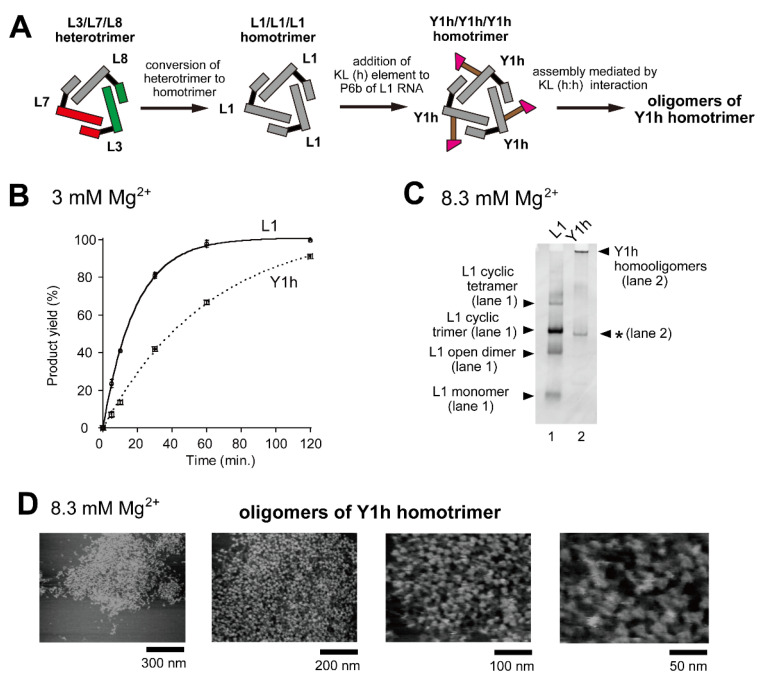
Oligomerization of a ribozyme cyclic homotrimer through a homo-KL (h:h) interaction. (**A**) Modular engineering to simplify the L3/L7/L8 heterotrimer to the corresponding homotrimer L1/L1/L1 followed by introduction of the KL (h) element to yield the Y1h/Y1hY1h homotrimer. (**B**) Substrate cleavage reactions by the parent ribozyme trimer, its derivatives, and their components. The five reactions contained 0.90 μM substrate-a. In the reactions with three RNA components, the concentration of L1 RNA or Y1h RNA was 0.90 μM. Reactions were carried out in the presence of 3 mM Mg^2+^. (**C**) EMSA of the L1 homotrimer and Y1h homotrimer in Tris-borate buffer (pH 8.3) containing 8.3 mM Mg^2+^. In each lane, the concentration of the RNA component was 1.2 μM. An asterisk indicates an unidentified oligomer of Y1h RNA. (**D**) AFM imaging of oligomeric states of the Y1h homotrimer. AFM was performed in Tris-borate buffer (pH 8.3) containing 8.3 mM Mg^2+^.

We designed and constructed ribozyme-assembly nanostructures using L3/L7/L8 and L3/L24/L25 heterotrimers (Figure 1D) as unit oligomers. The use of hetero-KL (α:α′ and β:β′) interactions (Figure S1) enabled preferential oligomerization of the unit trimers to induce cyclic tetramerization (Figure 6A and Figure 7D). As a strategy to further expand ribozyme assembly induced by the homo-KL (h:h) interaction (Y3h/Y7h/L8 system), we examined a unit trimer composed only of Y RNA (Y/Y/Y-type trimer). To simplify the composition of component RNAs, we altered the core trimer structure (L RNA trimer) from the heterotrimer system (L3/L7/L8) to a homotrimer (L1/L1/L1) system (Figure 8A). Homo-oligomerization of L1 RNA was shown to predominantly form a cyclic trimer, although a small amount of cyclic tetramer also formed as a minor assembly product [16]. To this L1 homotrimer system, we introduced the homo-KL (h) element to yield Y1h RNA (Figure 8A). The resulting homotrimer Y1h/Y1h/Y1h formed three homo-KL (h:h) interactions, and the extent of ribozyme trimer unit assembly was expected to increase significantly because each unit trimer has three KL elements (Figure 8A), and thus not only linear but also branched assembly became possible. Although the oligomerization may be less ordered and less controlled than that of a heterotrimer with hetero-KL interaction, we expected Y1h RNA to be capable of forming large oligomers of the RNA trimer units.

We first analyzed the catalytic activity of Y1h RNA to determine the effects of KL-dependent oligomerization of the L1 trimer (Figure 8B). In the presence of 3 mM Mg^2+^, Y1h RNA cleaved the substrate RNA (Figure 8B) but its activity (0.016 min^−1^) was 3.3-fold lower than that of L1 RNA (0.053 min^−1^). We then performed EMSA to confirm oligomerization of Y1h RNA. Y1h RNA did not migrate on nondenaturing polyacrylamide gel electrophoresis (PAGE), indicating the formation of Y1h RNA oligomers (Figure 8C, lane 2). Although EMSA indicated oligomerization of Y1h RNA, it yielded no information regarding the extent of its oligomerization.

To evaluate the extent of oligomerization directly, we analyzed oligomeric states of Y1h RNA by AFM in the presence of 8.3 mM Mg^2+^ (Figure 8D). The AFM images showed large assemblies of Y1h RNA which reached nearly 1000 nm in size, and the number of unit ribozyme trimers was too large to count (Figure 8D, left). It should be noted that the minor cyclic tetramer of component RNA was observed more frequently in the assembly of Y1h RNA than in cyclic oligomerization (predominantly cyclic trimerization) of L1 RNA (Figure 8D).

## 3. Discussion

In this study, we introduced oligomerization elements into cyclic trimers of the engineered *Tetrahymena* ribozyme (L RNAs) to expand the size and complexity of their oligomeric states. We added a stem-loop element for the KL interaction to the P6b element of L RNAs to yield a series of engineered L RNAs (termed Y RNAs). The ribozyme trimers with KL-mediated assembly ability formed dimeric states (Y/L/L-type unit trimers), linear oligomers (Y/Y/L-type unit trimers), and higher-order oligomers with branched oligomerization (Y/Y/Y-type unit trimers) depending on the number of Y RNA elements in the unit trimers. The assembly properties of the Y/Y/L-type unit trimers can be altered by changing the homo-KL (h:h) interaction to hetero-KL (α:α′) interaction. Linear oligomers of Y/Y/L-type unit trimers preferentially underwent ring-closing tetramerization to form cyclic tetramers when they had a hetero-L/L/L (L3/L7/L8 or L3/L24/L25) trimer core and a hetero-KL (α:α′ or β:β′) interaction. The resulting cyclic homotetramer of the cyclic heterotrimer consisted of eight Y RNAs and four L RNAs. Such ribozyme dodecamers cannot be analyzed by nondenaturing PAGE because they were too large to enter and migrate into the gel. The RNA remained stuck in the sample well, suggesting the formation of assemblies containing nine or more L/Y RNA components [28,29]. Direct measurement by AFM revealed their dodecameric assembly structures (Figure 6A and Figure 7D). In contrast to the preferential formation of discrete structures of the Y/Y/L-type heterotrimer with hetero-KL interactions, very large oligomers were observed in the Y/Y/Y-type homotrimer unit consisting of a homotrimer core (L1/L1/L1) with the homo-KL (h:h) interaction. AFM measurements revealed that assembly of the Y1h/Y1h/Y1h homotrimer formed large oligomers the size of which reached the micrometer order (Figure 8D).

The substrate cleavage activities of oligomers of the ribozyme trimers suggested that there was no functional advantage with regard to the enzymatic activity of the unit trimer upon oligomerization. In the case of oligomerization mediated by homo-KL interaction, the resulting oligomers (Y3h/Y7h/L8 and Y1h/Y1h/Y1h) had distinctly lower enzymatic activities than the parent L/L/L trimers (L3/L7/L8 and L1/L1/L1). The trimers formed using KL (h:h)-dependent oligomerization, which induced efficient but poorly controlled assembly of the ribozyme trimers and/or their components, showed reduced catalytic activity. Ribozyme trimer units in the KL (h:h)-mediated oligomeric states may have structural defects and/or be subject to structural strain. In contrast to ribozyme oligomers formed by homo-KL interactions, oligomerization mediated by hetero-KL (α:α′ or β:β′) interactions showed no significant negative effects on catalytic activity. This observation suggested that hetero-KL-mediated assembly may cause structural restrictions, which may be minimized by cyclic tetramerization in the Y/Y/L systems.

The ribozyme dodecamers identified in this study can be compared with the hemoglobin dodecamer and may be utilized as core modules to generate larger ribozyme complexes corresponding to giant hemoglobin formed through assembly of the hemoglobin dodecamer [2,6]. Y RNA still possessed the P9.2 structural element, which can be employed to install additional interacting motifs for further assembly. The P9.2 element was successfully employed as a pillar element for structural expansion of the ribozyme cyclic trimers and cyclic tetramers through face-to-face dimerization [29]. Introduction of the second KL interaction to P9.2 element to some monomer units in the ribozyme dodecamers may induce dimerization or even oligomerization of ribozyme dodecamers and thus yield giant oligomers with defined and discrete structures.

While the design and investigation of self-assembly of functional RNAs are important for their application in biotechnology and synthetic biology as RNA tools [13,14,15], self-assembly of ribozymes and other functional noncoding RNAs has also been suggested to have been involved in the evolution of the RNA world [31,32]. Condensation, phase separation, and related biophysical phenomena of RNA components would have occurred in primitive protocellular systems [33,34,35,36,37] prior to the establishment of systems compartmentalized by lipid bilayer membranes [38,39,40]. The present study suggested that oligomerization of L/Y/Y-type heterotrimers through the homo-KL (h:h) interaction induced relatively large but poorly controlled assembly of L/Y-RNA (Figure 3). The Y/Y/Y-type homotrimer (Y1h/Y1h/Y1h) with homo-KL (h:h) interaction also formed large and poorly controlled assemblies, which reached 1000 nm in size on AFM measurement (Figure 8D). It will be important to determine whether Y1h oligomers share some physical properties observed in condensed and/or phase-separated states of biomacromolecules. Coacervation and liquid–liquid phase separation (LLPS) containing RNA molecules have been reported in which ribozymes served as the RNA components [33,34,35,36,37]. In most previous examples, however, other macromolecular components, such as polypeptides and polysaccharides, were also required as essential components. Y1h-based large RNA assemblies represent interesting examples and/or precursors to generate ribozyme coacervates and ribozyme LLPS without polypeptides or polysaccharides. Such ribozyme assemblies could be interesting models for examining the emergence of protocellular states and structures in the RNA world.

## 4. Materials and Methods

### 4.1. Molecular Design

A model 3D structure of the full-length *Tetrahymena* group I ribozyme (PDB ID: 6WLS) and the crystal structures of coaxially stacked KL interactions of HIV-1 RNA (PDB ID: 1K9W and 2B8R) were used for construction of 3D models of L and Y RNAs and their oligomers (cyclic trimers of L and/or Y RNAs, KL-mediated dimers of Y RNAs and KL-mediated dimers of Y/L/L-type cyclic trimers). Molecular modeling was performed using Discovery Studio (BIOVIA, San Diego, CA, USA).

### 4.2. Plasmid Construction and RNA Preparation

The plasmid DNAs newly constructed in this study were made by PCR-based mutagenesis from plasmids reported previously [16,17]. Plasmids encoding sequences of L24 and L25 RNAs were derived from the plasmid encoding L1 RNA. Plasmids encoding sequences of Y1h RNA containing KL elements at P6b were derived from L1 RNA by replacing the loop at the end of the P6b element. Similarly, plasmids encoding sequences of other Y RNAs were also constructed from plasmids encoding sequences of the corresponding L RNAs. Subsequently, these plasmids were used as templates for PCR to amplify DNA fragments for in vitro transcription. The T7 promoter sequence was added by PCR with a sense primer containing the promoter sequence. Each in vitro transcription reaction was performed with T7 RNA polymerase for 4.5 h at 37 °C in 50 mM Tris-HCl (pH 7.5) buffer containing 15 mM Mg^2+^ and 1 mM of each nucleotide triphosphate. The solution was treated with DNase I for 30 min to digest the DNA template. The transcribed RNA was then purified by 6% denaturing PAGE. Labeling of RNA 3′-ends with BODIPY (boron-dipyrromethene)-fluorophore was performed according to the published procedure [41].

### 4.3. Electrophoretic Mobility Shift Assay (EMSA)

For assembly of each RNA structure, an aqueous solution of the appropriate set of RNAs was heated at 80 °C for 2.5 min. Subsequently, the incubation temperature was changed to 37 °C by adjusting the temperature setting on the block incubator. To this solution was added 10× concentrated folding buffer at 37 °C (final concentration 70 mM Tris-borate, pH 8.3, and 8.3 mM Mg(OAc)_2_ or 80 mM Tris-acetate, pH 7.5, and 17.5 mM Mg(OAc)_2_). The resulting mixtures were then incubated at 37 °C for 30 min for preparation of most of the RNA structures. An extended incubation period at 37 °C (1 h) was applied for formation of the homodimer of the Y3h/L7/L8 or L3/Y7h/L8 trimer. To prepare the heterodimer of the two trimers (Y3α/L7/L8 and Y3α′/L7/L8), each trimer was assembled separately and then mixed. The resulting solution was subsequently incubated at 37 °C for an additional 1 h. Each sample solution was kept at 4 °C for 30 min and then mixed with 6× loading buffer containing 0.1% xylene cyanol and 50% glycerol. Samples were electrophoresed on 5% polyacrylamide gels (acrylamide:bisacrylamide = 39:1) containing 70 mM Tris-borate (pH 8.3) or 80 mM Tris-acetate (pH 7.5) and a given concentration of Mg(OAc)_2_. The running buffer and the loading solution containing dye also possess the same concentration of Mg(OAc)_2_. Electrophoresis was performed at 4 °C, 200 V for the first 5 min and then 75 V for 12 h. To visualize nonlabeled RNA samples, the gels were treated with SYBR Green and then analyzed using a Pharos-FX fluoroimager (Bio-Rad, Hercules, CA, USA) or treated with ethidium bromide and visualized using a gel-documentation system (DS-430; BIO CRAFT, Tokyo, Japan). BODIPY-labeled RNA samples were visualized using a Pharos-FX fluoroimager (Bio-Rad). It should be noted that we initially performed EMSA using various concentrations of RNA and Mg^2+^. By analyzing the gel bands corresponding to the anticipated nanostructures, residual monomer units, and undesired oligomers, we established the optimal conditions for EMSA for each set of individual RNAs.

### 4.4. Ribozyme Activity Assay

Appropriate sets of aqueous RNA solutions (final concentration of each RNA component: 0.15–0.9 μM) were heated separately at 80 °C for 5 min. Subsequently, the incubation temperature was changed to 37 °C by adjusting the temperature setting on the block incubator. The RNA components were then mixed at 37 °C. The 10× concentrated reaction buffer (final concentrations: 30 mM Tris-HCl, pH 7.5, and 3 mM or 3.75 mM MgCl_2_) and 2 mM guanosine triphosphate (final concentration: 0.2 mM) were added to the RNA solutions and incubated at 37 °C for 30 min. Ribozyme reactions were initiated by introducing fluorophore-labeled substrate-a into the reaction mixture at a final concentration of 0.9 μM, and the reaction was allowed to continue at 37 °C. At specific time points, aliquots were taken and mixed with 1.5 volumes of stop solution containing 0.1% xylene cyanol, 100 mM EDTA, and 75% formamide to effectively halt the reaction. The resulting mixtures were electrophoresed on 15% polyacrylamide gels containing 7 M urea. Reactions of FAM- and TAMRA-labeled substrate RNAs were visualized using a Pharos-FX fluoroimager (Bio-Rad) and a gel-documentation system (DS-430; BIO CRAFT), respectively. Quantification of visualized gel bands was carried out using Quantity One software version 4.5 (Bio-Rad, Hercules, CA, USA). In the preliminary activity assay with L1 RNA homotrimer to compare FAM (Carboxyfluorescein)- and TAMRA (Carboxytetramethylrhodamine)-labeled RNA substrates, the two substrate RNAs provided nearly identical results (Figure S7). All assays were repeated at least twice. The mean values are shown in the figures, in which error bars represent the range from the minimal to the maximal values.

### 4.5. Atomic Force Microscopy (AFM)

AFM was carried out with a high-speed instrument (Nano Live Vision; RIBM, Tsukuba, Japan). Assembly of RNA components in buffer (final concentration: 70 mM Tris-borate, pH 8.3, and 8.3 mM Mg(OAc)_2_ or 80 mM Tris-acetate, pH 7.5, and 17.5 mM Mg(OAc)_2_) was performed according to the protocol for EMSA. Total concentrations of RNAs in the solution were then adjusted to ~80 nM by addition of sample loading buffer (70 mM Tris-borate, pH 8.3, and 8.3 mM Mg(OAc)_2_ or 80 mM Tris-acetate, pH 7.5, and 17.5 mM Mg(OAc)_2_). The sample solution (2 μL) was deposited onto a mica surface coated with 0.1% (3-aminopropyl)triethoxysilane and incubated for 5 min. The mica surface was then washed gently with sample loading buffer. The samples in the loading buffer solution were imaged at ambient temperature. A cantilever for High Speed AFM (Ultra-Short Cantilevers USC-F1.2-k0.15-10; NanoWorld, Neuchatel, Switzerland) with a spring constant of 0.1–0.2 N/m and a resonant frequency of 400–1000 kHz was employed. AFM imaging was performed at a scan rate of 0.2 fps.

## 5. Conclusions

We previously constructed and characterized engineered derivatives of the *Tetrahymena* ribozyme that formed cyclic trimers. These cyclic ribozyme trimers can serve as unit structures to develop larger and more complex assemblies. Oligomerization of the cyclic ribozyme trimers in this study preferentially yielded cyclic tetramers. Although further improvements of assembly efficiency and selectivity are needed, the resulting ribozyme dodecamer represents a promising platform for the development of larger ribozyme nanostructures that may correspond to giant hemoglobin consisting of 144 globin subunits and can be dissected into dodecamers of dodecamers. We also produced large and poorly ordered assemblies of ribozyme trimers, which reached 1000 nm in size. Such large ribozyme oligomers may represent possible protocellular nanostructures that may have emerged in the RNA world.

## Data Availability

Not applicable.

## Supplementary Materials

The following supporting information can be downloaded at: https://www.mdpi.com/article/10.3390/molecules28186465/s1, Figure S1: Scheme of stepwise modular design toward a cyclic tetramer of the ribozyme cyclic trimer; Figure S2: Secondary structures of L3 RNA and Y RNAs and assembly of Y RNAs; Figure S3: Sequences and three-dimensional structures of HIV DIS kissing loop interactions; Figure S4: EMSA of the L3, L7, L8, and Y3h RNAs in the absence of Mg2+; Figure S5: Secondary structures of one L RNA and five Y RNAs; Figure S6: EMSA of RNA components to form oligomers of the cyclic trimer in Tris-borate buffer (pH 8.3) containing 8.3 mM Mg2+; Figure S7: Effects of fluorophore molecules (FAM and TAMRA) attached to the 5′-end of the substrate RNA on the ribozyme-catalyzed cleavage reaction.

## Sample Availability

Samples of the compounds are not available.
