# Supplementary material for: Structural Expansion of Catalytic RNA Nanostructures through Oligomerization of a Cyclic Trimer of Engineered Ribozymes
Source: Molecules. 2023 Sep 6;28(18):6465. doi: 10.3390/molecules28186465 (PMC10535472; doi:10.3390/molecules28186465)
Supplement: Supplementary file 1 [file molecules-28-06465-s001.zip › molecules-2570643-supplementary.pdf]

Supplementary Materials

For

Structural Expansion of Catalytic RNA Nanostructures through Oligomerization of a Cyclic Trimer of Engineered Ribozymes

Mst. Ayesha Siddika [1], Hiroki Oi [2], Kumi Hidaka [3], Hiroshi Sugiyama [4], Masayuki Endo [4,5], Shigeyoshi Matsumura [1,2] and Yoshiya Ikawa [1,2,*]

[1] Graduate School of Innovative Life Science, University of Toyama, Toyama 930-8555, Toyama, Japan; smatsumu@sci.u-toyama.ac.jp (S.M.)
[2] Department of Chemistry, Graduate School of Science and Engineering, University of Toyama, Toyama 930-8555, Toyama, Japan
[3] Department of Chemistry, Graduate School of Science, Kyoto University, Kyoto 606-8501, Kyoto, Japan
[4] Institute for Integrated Cell-Material Sciences, Kyoto University, Kyoto 606-8501, Kyoto, Japan; sugiyama.hiroshi.3s@kyoto-u.ac.jp (H.S.); endo@kansai-u.ac.jp (M.E.)
[5] Organization for Research and Development of Innovative Science and Technology, Kansai University, Suita 564-8680, Osaka, Japan
* Correspondence: yikawa@sci.u-toyama.ac.jp

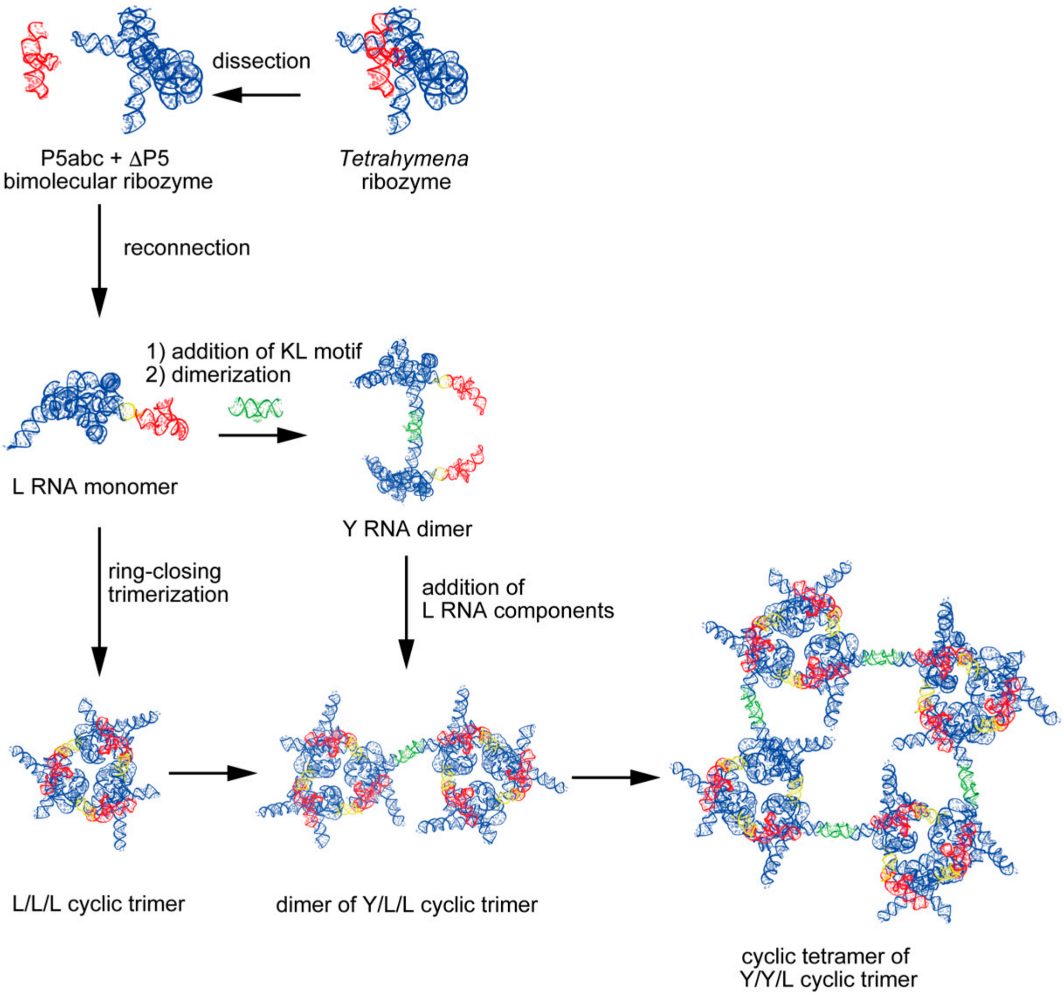

Figure S1. Scheme of stepwise modular design toward a cyclic tetramer of the ribozyme cyclic trimer.

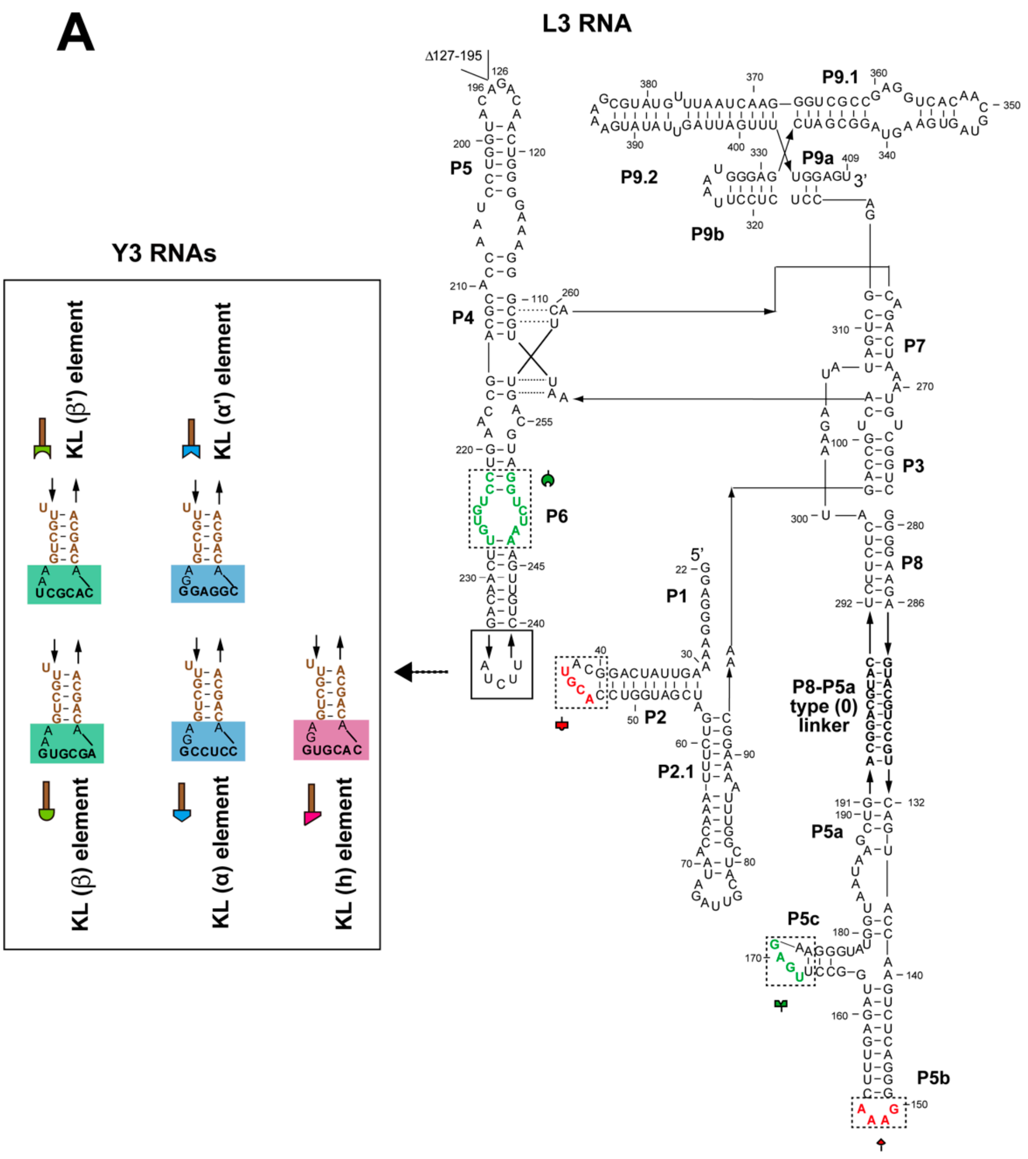
A
L3 RNA
Y3 RNAs
KL (β') element
KL (α') element
KL (β) element
KL (α) element
KL (h) element
P1
P2
P2.1
P3
P4
P5
P5a
P5b
P5c
P6
P7
P8
P9a
P9b
P9.1
P9.2
P8-P5a type (0) linker
Δ127-195
5'
3'

Figure S2.

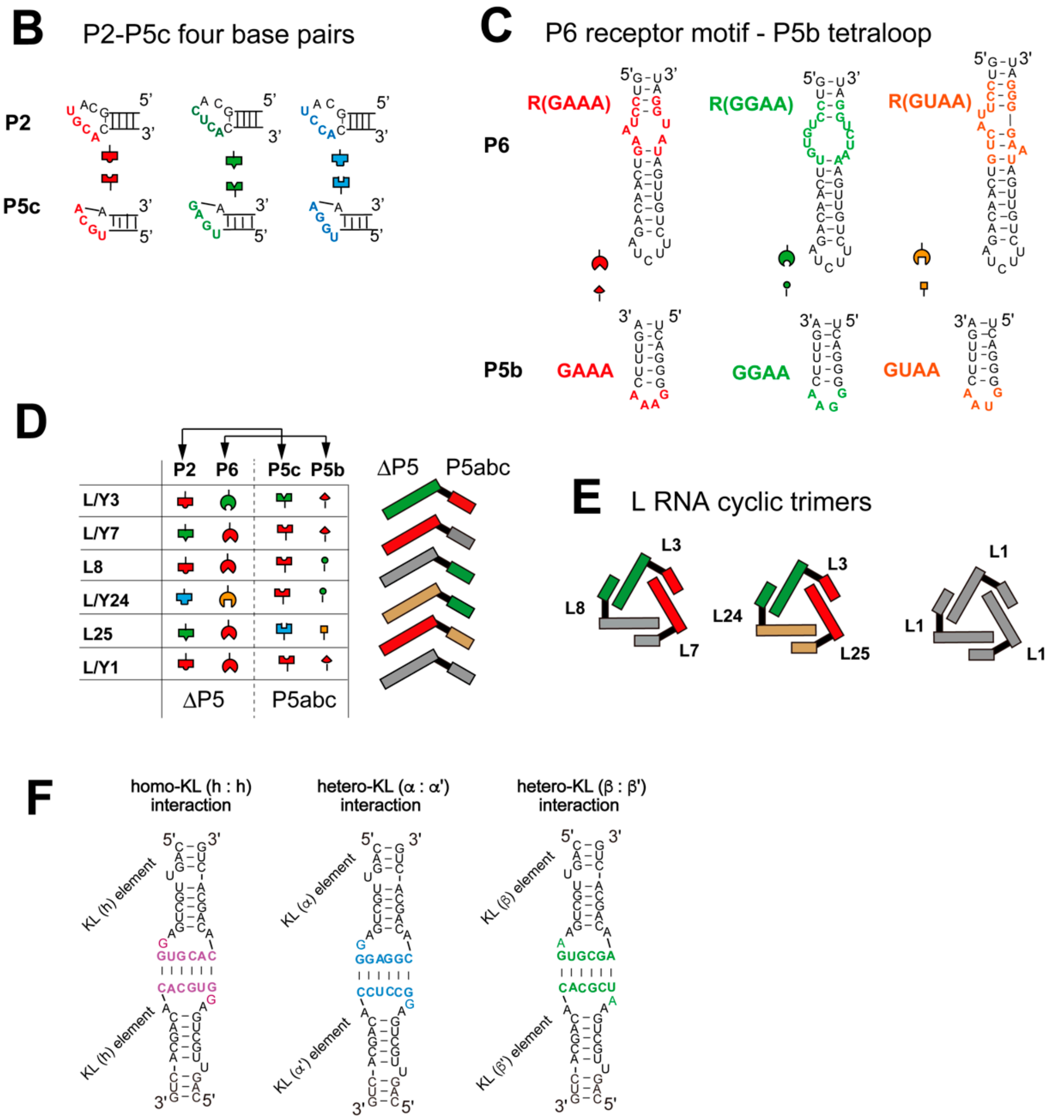

Figure S2. Secondary structures of L3 RNA and Y RNAs and assembly of Y RNAs.

A) Secondary structure of L3 RNA with the type (0) linker connecting P8 and P5a elements. The P1 element was designed to recognize the substrate-a RNA. Boxes with broken lines indicate modularly replaceable RNA elements to derive variants from the parent L1 RNA. Nucleotides shown in red constitute RNA motifs responsible for the intermodule tertiary interactions and present in the *Tetrahymena* ribozyme and in Y1 RNA. Nucleotides shown in green and black boldface indicate RNA elements artificially introduced for modular engineering. Five Y3 RNAs were derived from L3 RNA by replacing the P6b terminal 5′-AUCUU-3′ loop with the kissing loop (KL) elements shown in the box with solid lines. Numbers 1–21, 127–131, 192–195, and 287–291 are therefore absent in the secondary structure of L1 RNA. Numbers 132–191 (corresponding to the P5abc module) are inserted between 286 and 292 (corresponding to the P8 element). No numbers were assigned to the P8–P5a linker and the KL elements in P6b.

B) Three pairs of P2–P5c interactions to produce orthogonal ΔP5/P5abc interfaces.

C) Three pairs of P6–P5b interactions to produce orthogonal ΔP5/P5abc interfaces.

D) Tertiary interactions constituting interfaces between the ΔP5 ribozyme module and the P5abc module (left). L RNAs possessing distinct sets of ΔP5 and P5abc modules (right). Cognate pairs of ΔP5 and P5abc modules to form matched ΔP5/P5abc interfaces are shown in the same colors.

E) Three ribozyme cyclic trimers used in this study. The L3, L7, and L8 trio and the L3, L24, and L25 trio formed heterotrimers, while L1 RNA formed a homotrimer.

F) Three kissing loop (KL) interactions used in this study. The KL (h) element formed a homo-KL interaction, whereas the KL (α or β) element formed a hetero-KL interaction with the KL (α′ or β′) element, respectively.

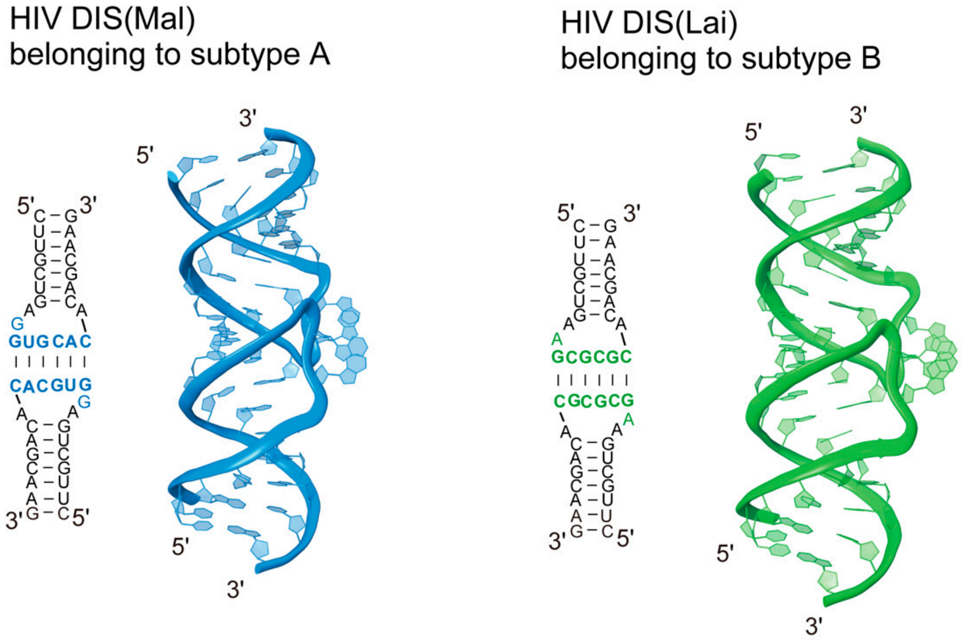

Figure S3. Sequences and three-dimensional structures of HIV DIS kissing loop interactions.

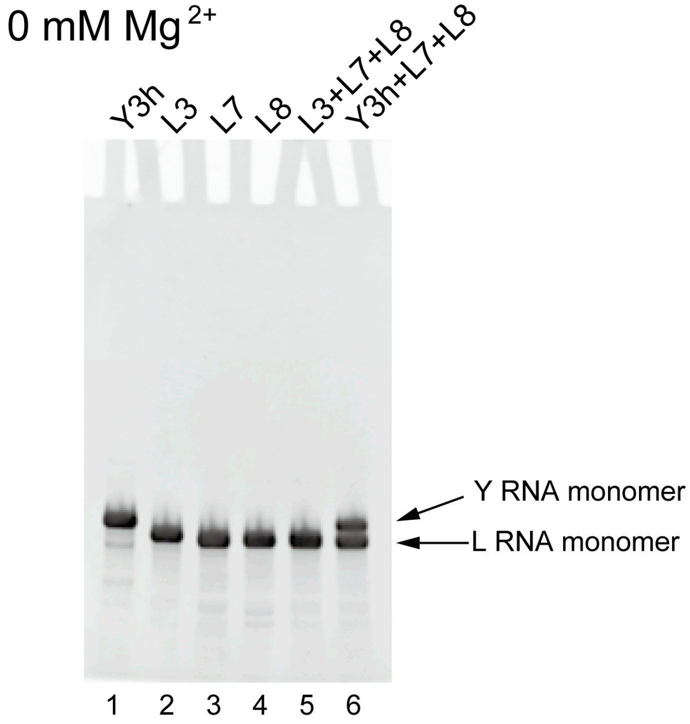

Figure S4. EMSA of the L3, L7, L8, and Y3h RNAs in the absence of Mg^{2+}.
Electrophoresis was carried out with Tris-borate buffer (pH 8.3). In lanes 1–4, the concentration of the RNA component was 1.2 μM. In lanes 5 and 6, the concentration of each RNA component was 0.40 μM.

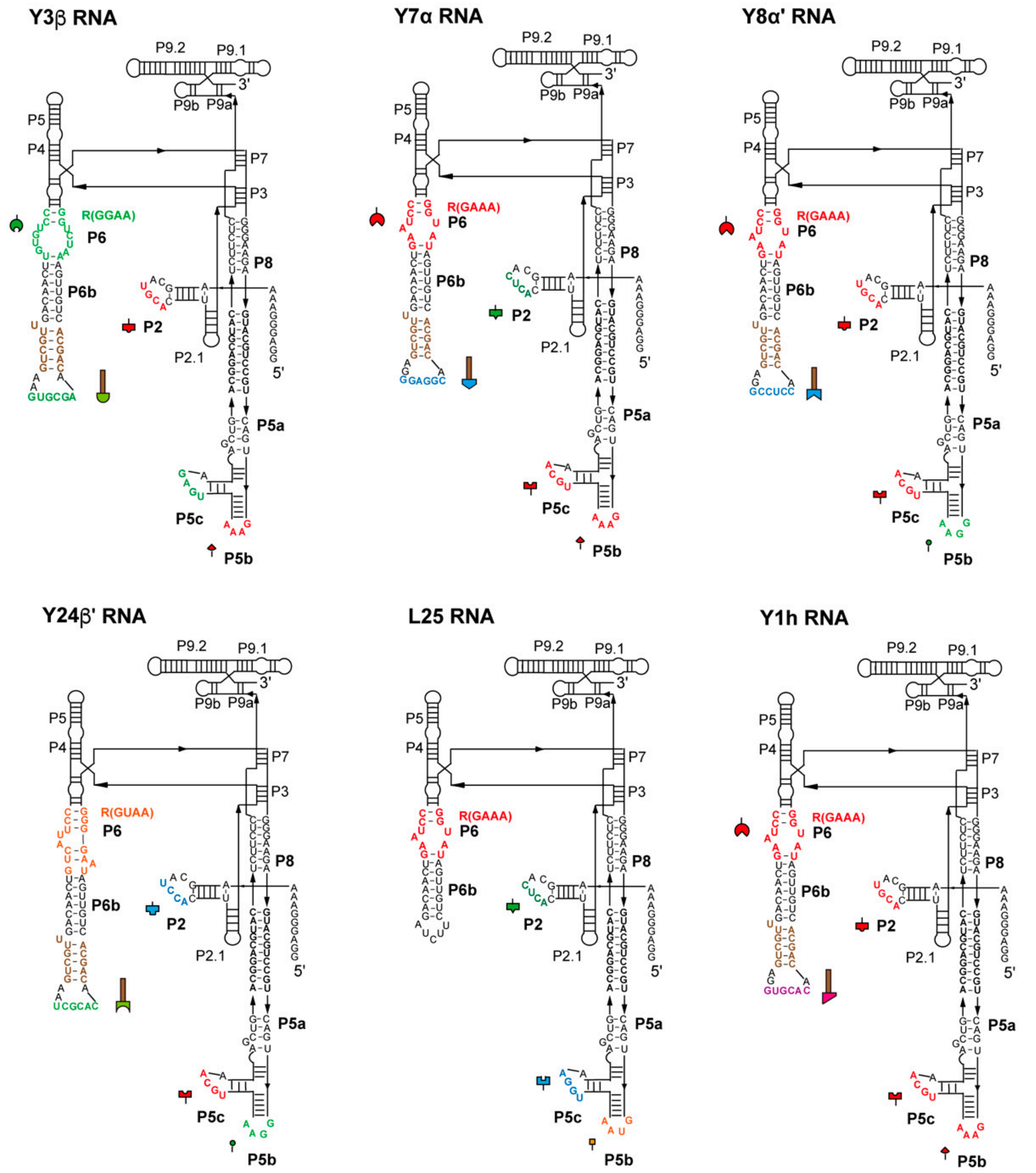

Figure S5. Secondary structures of one L RNA and five Y RNAs. Each had a distinct combination of RNA motifs and KL element.

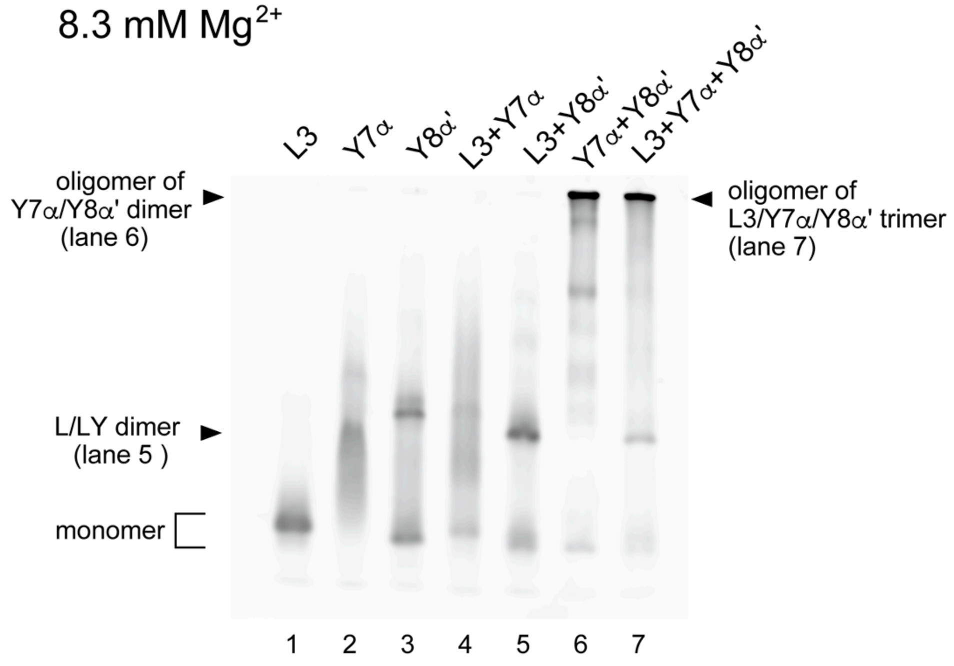

Figure S6. EMSA of RNA components to form oligomers of the cyclic trimer in Tris-borate buffer (pH 8.3) containing 8.3 mM Mg^{2+}.

In lanes 1–3, the concentration of the RNA component was 1.2 μM. In lanes 4 and 5, the concentration of each RNA component was 0.60 μM. In lanes 6 and 7, the concentration of each RNA component was 0.40 μM.

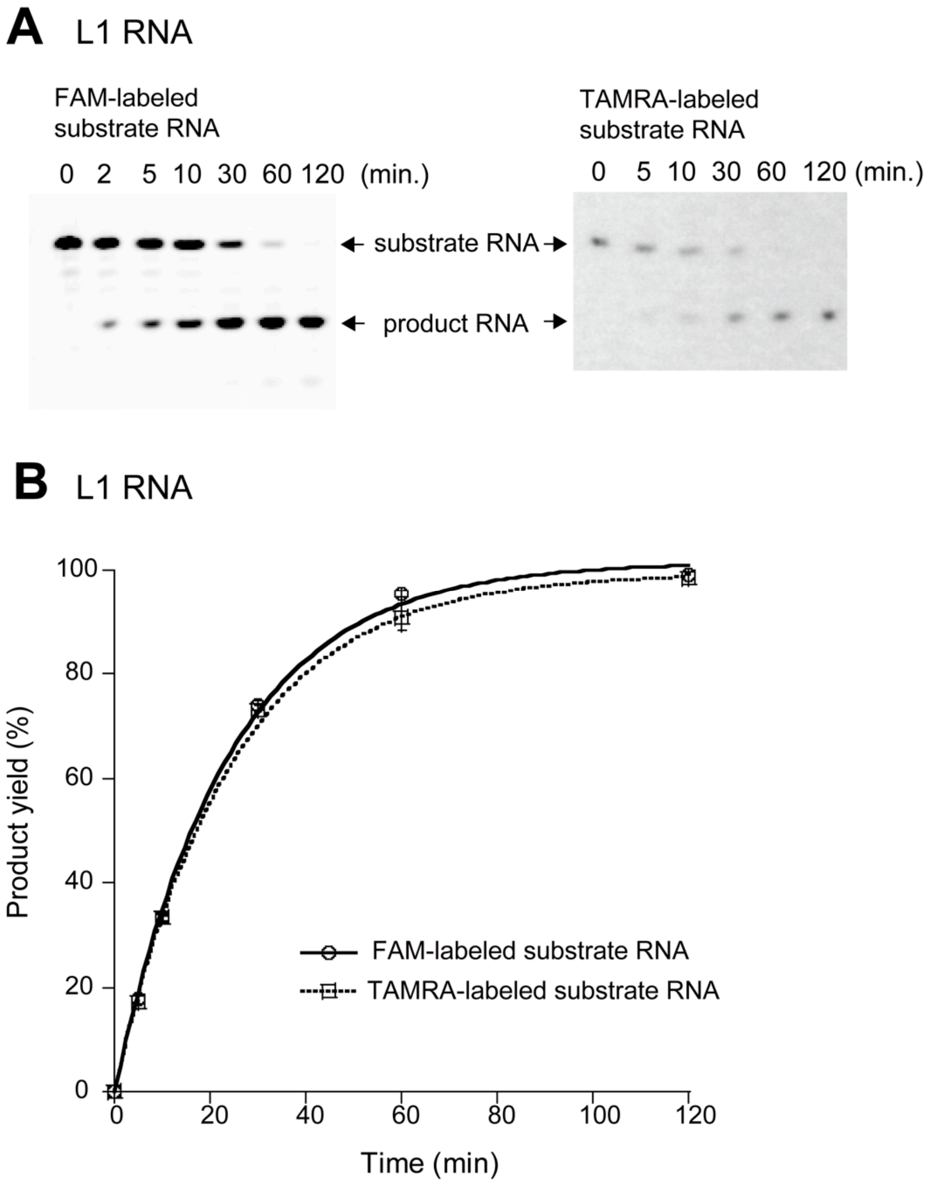

Figure S7. Effects of fluorophore molecules (FAM and TAMRA) attached to the 5′-end of the substrate RNA on the ribozyme-catalyzed cleavage reaction.

The concentration of L1 RNA was 0.50 μM. The concentration of the substrate RNA was 0.50 μM. Reactions were carried out in the presence of 3 mM Mg^{2+}. Gel-images and time-courses of the reactions are shown in Figures S7A and S7B respectively.
